# Simultaneous Content Determination of Mono-, Di-, and Fructo-oligosaccharides in Citrus Fruit Juices Using an FTIR-PLS Method Based on Selected Absorption Bands

**DOI:** 10.1155/2024/9265590

**Published:** 2024-01-10

**Authors:** Yurika Yajima, Hideyuki Wakabayashi, Ken-ichiro Suehara, Takaharu Kameoka, Atsushi Hashimoto

**Affiliations:** ^1^Institute for Future Beverages, Research & Development Division, Kirin Holdings Company, Limited, 1-17-1 Namamugi, Tsurumi-ku, Yokohama, Kanagawa 230-8628, Japan; ^2^Graduate School of Regional Innovation Studies, Mie University, 1577 Kurimamachiya-cho, Tsu, Mie 514-8507, Japan; ^3^Graduate School of Bioresources, Mie University, 1577 Kurimamachiya-cho, Tsu, Mie 514-8507, Japan

## Abstract

A quantification method was developed to determine the sugar components, either following addition or enzymatic treatment, in citrus fruit juices containing additional fructo-oligosaccharides using midinfrared spectroscopy. For the quantification, we compared the results obtained by applying the simultaneous equation method, which uses very little wavenumber information, and the partial least squares (PLS) regression method, which requires a lot of wavenumber information. In order to prevent overfitting in the PLS method, we concentrated on reducing the amount of spectral data used in the analysis. The corresponding FTIR-PLS method led to an accurate quantification of the sugar contents, even in enzymatically treated orange juices with complicated compositions. The spectral data used for model calibration were significantly reduced by focusing on the absorption and assignment information of the sugar components. The RMSEs of Glc, Fru, Suc, GF2, and GF3 in enzyme-treated orange juice before and after spectral data reduction were 0.50, 0.46, 0.61, 0.74, and 0.61 g/L and 0.51, 0.49, 0.73, 0.86, and 0.61 g/L, respectively. The developed method could be easily implemented for practical applications, using a simple measuring instrument since only absorption information at the limited absorption bands is required.

## 1. Introduction

In recent years, health consciousness has risen, and more consumers are becoming interested in the nutritional components contained in foods. In particular, the need for sweeteners has diversified, and it has been reported that, although saccharides such as mono- and disaccharides are good energy sources, their excessive consumption increases the risk of obesity and lifestyle-related diseases such as diabetes mellitus [1]. The World Health Organization (WHO) strongly recommends reducing sugar intake [2]; however, it has not yet been reduced to the target value. Many consumers actively pursue the intake of oligosaccharides, which have been confirmed to induce various physiological effects, such as improvement in blood lipid levels, growth stimulation of Bifidobacteria, indigestibility, noncariogenicity, and anticariogenicity [3, 4].

Soft drinks with no added sugar often contain either sweeteners, such as acesulfame potassium or aspartame, or oligosaccharides as functional ingredients, and are readily available to buy. Fruit and vegetable juices contain large amounts of functional ingredients, such as vitamins, polyphenols, and saccharides, including mono- and disaccharides. Because the raw materials themselves contain saccharides, the mainstream method to reduce the sugar content in fruit juice products is to reduce the amount of fruit juice itself, for example, by producing fruit drinks containing 50% of fruit juice. Unfortunately, this method dilutes the beneficial functional ingredients derived from the raw materials, such as *β*-cryptoxanthin and hesperidin in the case of citrus fruits, which have been reported to maintain bone health [5] and improve blood pressure [6] and blood lipids [7]. In order to reduce the content of saccharides while maintaining a 100% concentration of juice, those can be converted to other products, such as oligosaccharides, organic acids, and alcohols, using technologies such as enzymatic treatment [8] and fermentation [9, 10]. However, time-dependent changes in components may occur during enzymatic treatment and fermentation, and since saccharides are easily affected by the reaction time and temperature, it is necessary to determine the saccharide composition in a timely manner for stable production.

Refractive index and volumetric measurements are used industrially to analyze the saccharides in foods; however, these methods measure all sugars present within a sample, while it is difficult to separate and quantify individual sugar components. This can nonetheless be achieved by using an enzymatic method [11, 12] and high-performance liquid chromatography (HPLC) [13]; however, the specificity of the enzymatic method may be insufficient when saccharides and oligosaccharides are mixed. Although HPLC can separate and quantify sugars with high precision, the method can present shortcomings at the manufacturing sites due to requirements such as pretreatment and analysis time, complexity of equipment maintenance, and use of organic chemicals.

Spectroscopy is an analytical method in which information is obtained by applying light of a specific wavelength to a substance and measuring the corresponding light transmitted or reflected. Infrared spectroscopy analyzes the vibrational modes of functional groups in a sample and quantifies its components. Since infrared spectroscopy requires almost no sample pretreatment and is rapid, cost effective, simple, and easy to implement in manufacturing plants, it is an excellent alternative technique to analyze the sugar concentration in food samples. In particular, Fourier transform infrared spectroscopy (FTIR) combined with the attenuated total reflection (ATR) method can be used to quantify sugars in liquid foods such as juices and soft drinks. Kameoka et al. successfully quantified individual components from a sugar solution or orange juice using only the absorbance at the wavenumber of the respective sugar component, combined with simultaneous equations based on their spectral additivity and Bouguer-Beer's law [14]. In other studies, the method has been increasingly used in combination with principal component regression (PCR) and/or partial least squares (PLS) regression analysis to produce highly accurate, multicomponent, and quantitative analyses of sugars in syrups [15], sugarcane juices [16–18], fruit juices [19–21], wine [22], honey [23–27], raw orange fruit [28], and cow milk [29]. Furthermore, it has been shown that the concentration of each sugar component can be quantified even in aqueous solutions and fruit juices containing oligosaccharides [18, 30]. PLS typically uses the full spectrum to predict the components in a mixture; however, inadvertent use of wavenumber information can cause considerable noise and irrelevant information that not only risks overfitting the predictive model but also decreases the generalization performance of the model [31–33]. As a countermeasure, it has been reported that a careful selection of wavelength ranges in multicomponent analysis can eliminate spectral ranges without information about the component, thereby improving accuracy [34, 35]. Simulated annealing and genetic algorithms [36] have been studied as methods of selecting wavenumbers; however, because the optimization of these methods is based on mechanical calculations, absorption bands associated with the structure of the contained components may not be detected, which can lead to large errors in juice samples, which are complex natural products.

The purpose of this study is to quantify the sugar components in fruit juices containing multiple oligosaccharides, which are similar in structure to the endogenous sugar content, using midinfrared spectroscopy. At first, we applied the simultaneous equation method (SE method) [14] to quantify the sugar components in enzyme-treated orange juice with a complicated sugar composition. The SE method uses very little wavenumber information for quantification. The risks of overfitting can be then avoided but may not be suitable for complex system. On the other hand, SE was combined with the PLS method, which uses many wavenumber information to confirm the sugars in orange juice. Furthermore, in order to prevent overfitting in the PLS method, we studied how to reduce the amount of spectral data used in the analysis. To narrow down the wavenumbers, we focused on the absorption and assignment information of the sugar components. We used two types of juices in this study: orange juice, in which fructo-oligosaccharides were enhanced by enzymatic treatment with fructosyltransferase to reduce the content of endogenous sucrose, and citrus juice in which fructo-oligosaccharide preparations were added.

## 2. Materials and Methods

### 2.1. Sugar Solutions

Sugars generally contained in citrus juice, including the monosaccharide glucose and fructose as well as the disaccharide sucrose (GF), were used. In addition, 1-kestose (GF2), nystose (GF3), and 4F-fructofuranosyl nystose (GF4), which are fructo-oligosaccharides consisting of *β*-(1,2)-linked fructoses attached to the fructose residues of sucrose, were used. The reagents were manufactured by FUJIFILM Wako Pure Chemicals (Japan). Solutions of mono- and disaccharides at ~120 g/L and fructo-oligosaccharides at ~80 g/L were prepared by dissolving them in distilled water.

### 2.2. Enzymatically Treated Citrus Juice Samples

Concentrated Valencia orange juice, made using oranges from different areas of Brazil and Spain, was enzymatically treated with *β*-fructosyltransferase (Shin Nihon Chemical Co., Ltd.), which reduces sucrose to fructo-oligosaccharides by transfructosylation. This enzyme allows to maintain a good flavor because it does not change the pH after the reaction.

The concentrated orange juice was diluted to Brix 45 with ion-exchanged water before *β*-fructosyltransferase was added at 9000-45000 U/L, and the reactions proceeded at 25°C. The enzymatic reactions were stopped by heating for 10 min at 80°C by sampling at predetermined intervals (1, 2, 3, 4, and 6 h). HPLC analysis of the concentrated orange juice before enzymatic reaction showed the presence of Glc, Fru, Suc, GF2, and GF3 at concentrations of 18.7, 20.7, 41.9, 0.0, and 0.0 g/L, respectively. The juice was then used to produce 75 samples, diluted to Brix 11 with ion-exchanged water, which were enzymatically treated with varying amounts of enzyme at different reaction times. A sample treated with 9000 U/L of enzyme for 6 h contained 30.3, 18.7, 11.1, 12.1, and 2.3 g/L of Glc, Fru, Suc, GF2, and GF3, respectively.

### 2.3. Citrus Juice Samples

Fructo-oligosaccharides were added to concentrated citrus juice varieties in order to regulate the intestinal function due to their effect on the growth of Bifidobacteria. The citrus juice varieties used were as follows: Valencia orange from different areas of Brazil and Spain, mandarin orange from Israel, white grapefruit and ruby grapefruit from the USA, and citrus unshiu from Korea. Two fructo-oligosaccharide formulations (Meioligo P and G, Meiji Food Materia Co., Ltd., Japan) with different compositions that are commonly used in food applications were used. Concentrated fruit juice was diluted to the equivalent of straight fruit juice with ion-exchanged water, and then, 18 samples of the citrus juice varieties were prepared with different concentrations of the fructo-oligosaccharide formulations ranging from 12 to 30 g/L (Table 1). For example, Valencia orange sample A was mixed with Meioligo P (12.0 g/L), resulting in Glc, Fru, Suc, GF2, GF3, and GF4 concentrations ranging from 18.7, 20.7, 41.9, 0.0, 0.0, and 0.0 g/L to 18.5, 21.6, 41.3, 5.0, 6.4, and 1.0 g/L, respectively.

### 2.4. Spectral Acquisition and Data Processing

The midinfrared absorbance spectra of the sugar solutions and fruit juice samples were measured using an FTIR spectrometer (Spectrum 400, PerkinElmer Inc.). The ATR method, which is less likely to be affected by water, was adopted for measurements using a 9-reflection ATR accessory of diamond crystal (Durasampl IR, Applied Systems). The measurement conditions for the spectra ranged from 4000 to 400 cm^−1^, with a resolution of 4 cm^−1^, and integration time of 16 scans, while the data were recorded at 2 cm^−1^ intervals under room temperature, dry air conditions. Spectral data from some of the samples were used to transform the absorbance to secondary derivative values using the Savitzky-Golay method (17 points) [37].

### 2.5. Multivariate Data Analysis

In the SE method for the determination of sugars in orange juice samples, we attempted to quantify the sugars by using the infrared absorption spectral information at the wavenumbers characterizing each sugar component, in accordance with previous studies [14].

Unscrambler X (CAMO Software Inc.) was used in the PLS analysis. In order to calibrate each model to predict the concentration of glucose, fructose, sucrose, GF2, GF3, and GF4 contained in fruit juice, multivariate analysis (PLS-1) was performed using the absorbance or secondary derivative values as explanatory variables and the quantitative value from HPLC as response variables. The calibrated model was evaluated using root mean square error (RMSE) calculated by cross-validation. For the enzyme-treated orange juice sample, 75 samples were randomly divided into 75, 15, 5, and 3 groups and cross-validated for each pattern. In addition, the wavenumber used for the explanatory variables was refined based on the peak information of the IR absorption spectra of each sugar component.

### 2.6. HPLC Analysis

Sugar components were quantitatively analyzed using an HPLC (Prominence-i, Shimadzu Co., Ltd.) in which a liquid pump system, autosampler, and refractive index detector were integrated. The separation of the sugar components was performed with an amino column (YMC-Pack Polyamine II 250 × 4.6 mm, YMC Co., Ltd.) and an isocratic method using acetonitrile/water (2 : 1, *v*/*v*) at a constant flow of 1.0 mL/min at 30°C. All samples were diluted with distilled water in order to obtain a concentration of 10 g/L or less for each sugar component based on the values measured by using the Bx saccharimeter (RX-5000i, Atago Co.,Ltd.) as a reference, centrifuged, and filtered (0.45 *μ*m). Each sugar component was identified by comparing its retention time (Rt) with that of standard solutions, and quantitative values were calculated with reference to the calibration curve created in the range from 0.25 to 10 g/L.

## 3. Results and Discussion

### 3.1. Infrared Spectral Features

**Figure 1:**
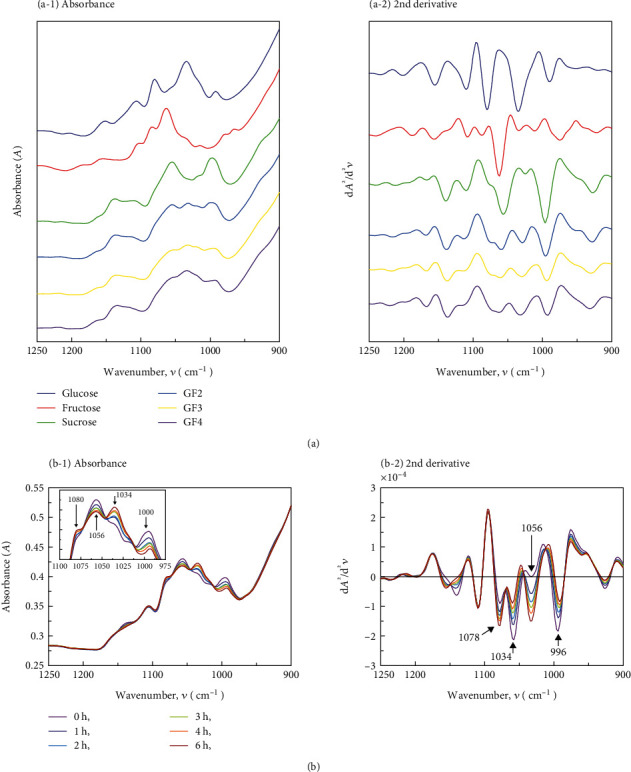
IR spectra of sugar solutions and enzymatically treated orange juice. An example of the spectra of orange juice over time from the start of enzyme treatment.

**Figure 2:**
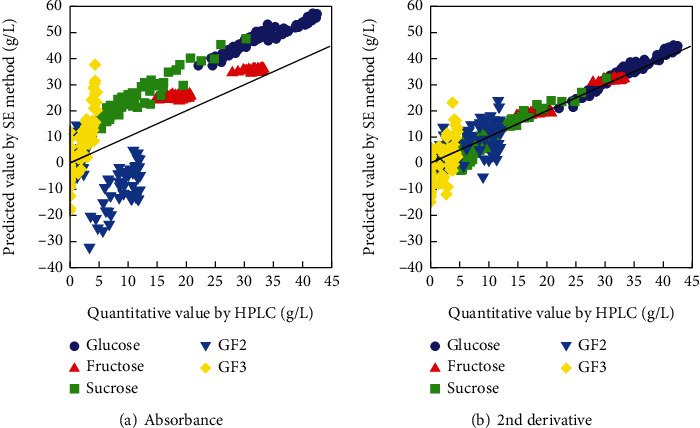
Relationship between predictive values using the SE method and quantitative values from HPLC of sugars in enzymatically treated orange juice.

Glucose, fructose, sucrose, GF2, GF3, and GF4 absorbed over a wide area; however, they showed an especially strong absorption pattern in the 1250 to 900 cm^−1^ region (fingerprint region) due to the stretching vibrational modes of C-O and C-C bonds of carbohydrates (Figure 1 (a-1, a-2)). Different absorption wavenumbers characterize each sugar component; strong absorption bands unique to glucose, fructose, and sucrose (monosaccharides and disaccharides with relatively simple structures) were confirmed. Among these, the absorption of C1-H bending around 1080 cm^−1^ and overlapping absorption of an ether CO and alcohol C-OH stretching around 1034 cm^−1^ were observed, which are the characteristic absorption bands of glucose [38, 39]. The overlapping absorption of an ether CO and alcohol C-OH stretching around 1064 cm^−1^ is the characteristic absorption band of fructose [39, 40]. The absorptions of an ether CO stretching around 1056 cm^−1^ and alcohol CO stretching (glycosidic linkage) around 1000 cm^−1^ are characteristic bands of sucrose [39, 41]. GF2, GF3, and GF4 are oligosaccharides with relatively complex structures; however, they have similar functional groups as their sugar constituents are the same. They produced a flat-spectrum pattern compared to mono- and disaccharides and displayed common absorption bands; absorption bands were also observed at around 1134, 1034, and 930 cm^−1^, which are characteristic of fructo-oligosaccharides [18, 30]. These three oligosaccharides can be identified by their spectral patterns in which the absorption intensity is slightly different due to the ratio of functional groups and their interaction caused by a difference in the degree of polymerization.

Figure 1 (b-1, b-2) shows the infrared spectra of orange juice over time during the enzymatic treatment process. The absorption at the wavenumber characteristic of sucrose, namely, around 1000 and 1056 cm^−1^ (around 996 and 1056 cm^−1^ in the second derivative), decreased in the enzymatically treated orange juice spectrum, while the peak shift was confirmed to be close to the peaks observed for GF2, GF3, and GF4. Moreover, the absorption increased over time at 1034 and 1080 cm^−1^ (for the second derivative at 1034 and 1078 cm^−1^), which correspond to the characteristic absorption wavenumbers of glucose (Figure 1 (b-1, b-2)). These changes were consistent with the changes in sugar composition measured using HPLC.

### 3.2. Quantification of Sugar Components in Enzyme-Treated Orange Juice

The concentration of sugars in 75 enzymatically treated orange juice samples, as measured by HPLC, provided ranges of glucose, fructose, sucrose, GF2, GF3, and GF4 of 18.4-42.5, 14.8-33.7, 0.6-41.9, 0.0-12.1, 0.0-4.6, and 0.0 g/L, respectively. GF4 was excluded from the components to be quantified as it was not detected in any of the enzymatically treated orange juice samples.

In order to quantify the sugar components using the SE method, a total of 18 wavenumbers, located at the peak of the absorption spectra, were extracted from each spectrum of glucose, fructose, sucrose, GF2, and GF3. Calibration curves for the 18 wavenumbers were then created for each of the 5 sugar components. The relationship between absorption and concentration at each wavenumber could be regressed on a straight line for all five types of sugar, with the absorbance of the spectrum of pure water as the intercept, so that the regression coefficient could be obtained. We selected 5 wavenumbers from the extracted 18 wavenumbers and, using a formula from previous studies [14, 42], substituted the absorbance of enzyme-treated orange juice at the 5 wavenumbers into the simultaneous equations, so that the concentration of each sugar in the enzyme-treated orange juice could be calculated. The absorbance of pure water was applied at each wavenumber to the section corresponding to the intercept in the formula. The relationship between the HPLC reference values and the results calculated from the absorption information at 1080, 1064, 998, 1034, and 1052 cm^−1^ is shown in Figure 2(a) as an example of the verification of wavenumber combinations that obtained the minimum RMSE. The RMSEs of glucose, fructose, sucrose, GF2, and GF3 were as large as 15.20, 6.13, 13.40, 16.42, and 11.19 g/L, respectively, and it was difficult to accurately quantify the concentration of sugar components by the SE method using absorbance.

We performed the same verification on the second derivative spectra in order to reduce the baseline shift and make it easier to observe the spectral characteristics of GF2 and GF3, which are flat in absorbance, making it difficult to extract the absorbed wavenumbers. The relationship between the HPLC reference values and the results calculated using information at wavelengths 990, 1156, 1056, 996, and 1060 cm^−1^ is shown in Figure 2(b). The RMSE was small. In the second derivative, the RMSEs for glucose, fructose, sucrose, GF2, and GF3 were 1.97, 1.31, 3.23, 5.36, and 7.02 g/L, respectively. Correlations with the HPLC values were detected in the high concentration region; however, it was difficult to accurately predict the sugar concentration in the low concentration region. GF2 and GF3 share similar unique characteristic wavenumbers; in addition, the regression coefficients at the selected wavenumbers are similar, making it particularly difficult to distinguish.

**Figure 3:**
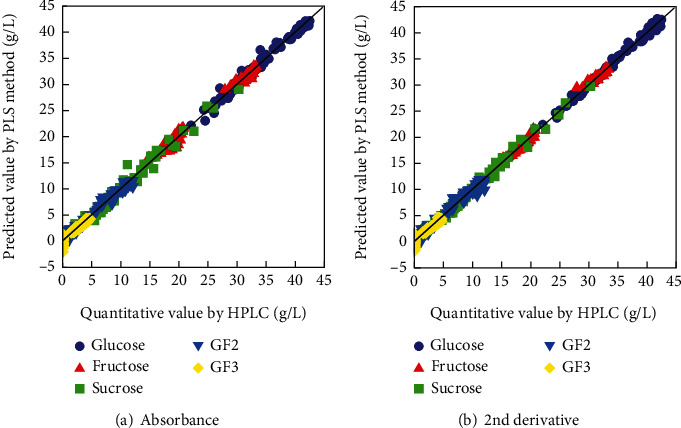
Relationship between predictive values using the PLS method and quantitative values from the HPLC of sugars in enzymatically treated orange juice. Absorbance: *R*^2^ of glucose, fructose, sucrose, GF2, and GF3 were 0.978, 0.989, 0.979, 0.978, and 0.883, respectively, and the number of factors was 2, 3, 5, 7, and 4, respectively. For the 2nd derivative: *R*^2^ of glucose, fructose, sucrose, GF2, and GF3 were 0.990, 0.995, 0.989, 0.968, and 0.879, respectively, and the number of factors was 3, 2, 4, 4, and 2, respectively.

Since sufficient accuracy could not be obtained by the SE method using information from only 5 wavenumbers for the 5 components, the analysis was performed using PLS, which is often used for component quantification based on infrared spectroscopy. A PLS-1 model was calibrated using infrared absorption information at all wavenumbers from 1250 to 900 cm^−1^ (176 points), with the sugar concentration measured by HPLC as the response variable. To determine how to evaluate the model, 75 enzyme-treated orange juice samples were randomly divided into 75, 15, 5, and 3 groups and cross-validated with each pattern. The results showed no dramatic differences in RMSE, *R*^2^, and number of factors among the 4 patterns; therefore, the model was calibrated by dividing into 3 groups of 25 samples and performing cross-validation. Figures 3(a) and 3(b) show the relationship between the results of concentration prediction using PLS regression and HPLC values. There is a high correlation between both values; the RMSEs of glucose, fructose, sucrose, GF2, and GF3 are 0.74, 0.66, 0.84, 0.62, and 0.60 g/L for absorbance and 0.50, 0.46, 0.61, 0.74, and 0.61 g/L for the second derivative, respectively. These findings suggest that the concentration prediction by PLS regression is suitable for samples containing multiple components with similar structures. As shown in the above results, the accuracy comparable to previous studies [21, 28, 30] was experimentally obtained for the content determination.

### 3.3. Effect of the Wavenumber Narrowing Step on Quantitative Accuracy

**Figure 4:**
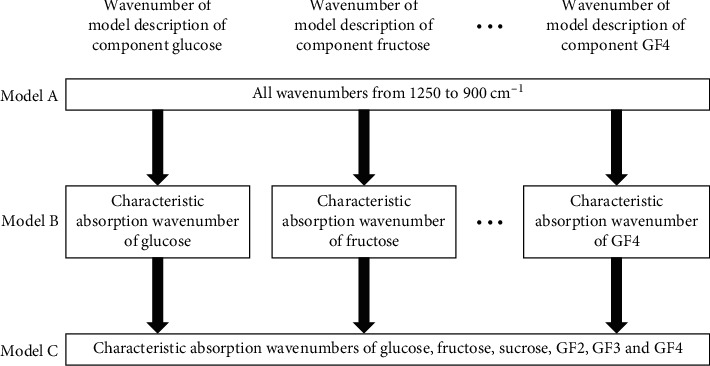
Steps for selecting wavenumbers as explanatory variables.

**Figure 5:**
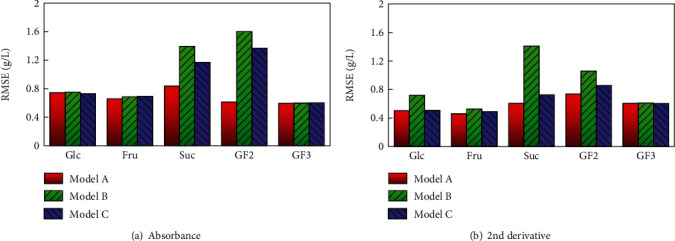
Effect of wavenumber selection step on RMSE of enzyme-treated orange juices.

In the previous section, we demonstrated that the information at 5 wavenumbers was insufficient for concentration quantification, but accurate quantification was possible when all wavenumbers in the fingerprint area were used, although the risks of overfitting would be unavoidable. In this section, we describe how the wavenumbers used for model calibration in the PLS method can be reduced. To this end, the characteristic peak wavenumbers within the range from 1250 to 900 cm^−1^ were deduced from the spectra of each sugar solution component and the absorption wavenumber information. The wavenumbers extracted from the absorbance spectra of glucose, fructose, sucrose, GF2, and GF3 were 8, 10, 10, 10, and 10 points, respectively. The second derivative values were 6, 7, 6, 9, and 11 points, respectively. Using these information, verification was performed in two stages (B, C) according to the wavenumber selection procedure shown in Figure 4. Model A is a model that uses infrared absorption information at all wavenumbers from 1250 to 900 cm^−1^ (176 points) calibrated in the previous section. Figures 5(a) and 5(b) show the RMSE of the predicted sugar concentration values obtained by cross-validation after selecting for wavenumbers in the patterns from A to C, with respect to the quantitative values measured by HPLC. In the wavenumber selection patterns of B and C, model B was calibrated with only a single component information and had lower accuracy compared to A without wavenumber selection. The accuracy was improved in model C, in which information from other components was added to B, so that the accuracy was improved and was close to the accuracy of model A. It can be assumed that, due to the presence and influence of other sugar components, information from one component is insufficient in predicting concentration and accuracy will decrease when using a limited spectrum. In particular, the errors calculated from the quantitative values measured by HPLC increased with low concentrations of sucrose, and it was confirmed that information on other components was necessary for accurate concentration prediction. It was considered that the presence of enzymatic products had a great influence on the measurements because the sample with reduced sucrose contained large amounts of GF2 and GF3. GF2 and GF3 have similar structures and generally have similar absorbance wavenumber bands; therefore, the use of only a single component information was insufficient because many common wavenumbers were shared. It should be possible to perform model calibrations with only the absorbance information at limited wavenumbers by appropriately inserting the information of other component sugars contained in the sample. Our results indicate that, by focusing on the peak wavenumber in the spectrum of all the components contained in the sample, it is possible to narrow down the wavenumber associated with the contained component; therefore, the infrared spectroscopic information of that wavenumber may be sufficient for accurate concentration determination. In general, it is difficult to use the spectral information observed on the near-infrared (NIR) region for determining the wavenumber associated with the contained component. The narrowing method of model C proposed in this study can also be applied to NIR spectroscopic methods by associating the fundamental vibrations of the contained components with the wavenumber range in which overtones and combination bands are observed. However, it may be difficult to distinguish between fructo-oligosaccharide types with NIR spectroscopy although it is relatively easy to develop a simple device compared to the FT-IR spectroscopic method. It would be desirable to properly use infrared spectroscopy to monitor the enzymatic reaction during the production process of enzyme-treated juices, while applying near-infrared spectroscopy only to check the sugar components in the final product. We will also study the application of the NIR spectroscopic method to investigate the enzyme-treated juice in the future.

### 3.4. Application to Citrus Fruit Juices with Added Fructo-oligosaccharides

**Figure 6:**
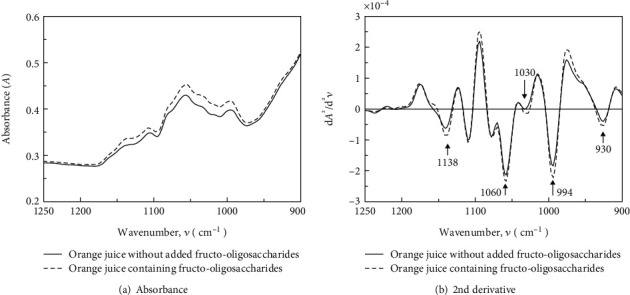
IR spectra of citrus juice samples.

It is generally known that GF2, GF3, and GF4 are produced as fructo-oligosaccharides in the enzymatic reaction with fructosyltransferase [30], although GF4 was not produced in the enzyme-treated orange juice considered in this study. We verified the concentration quantification of component sugars using the PLS method for fruit juices containing GF2, GF3, and GF4 by adding fructo-oligosaccharide preparations to various fruit juices. Figures 6(a) and 6(b) show the infrared spectra before and after the addition of fructo-oligosaccharide preparations to orange juice samples. Comparing the second derivative spectra, the orange juice samples containing fructo-oligosaccharides showed strong peaks around 930, 994, 1030, 1060, and 1138 cm^−1^, which are characteristic of the second derivative spectra of GF2, GF3, and GF4. Therefore, the presence of the fructo-oligosaccharide preparation was confirmed by infrared spectroscopy. Table 1 shows the results of HPLC quantification of the sugar components in the citrus fruit juices containing fructo-oligosaccharides. Although the sample set had various ratios of each sugar component, the ratios of GF4 to GF2 and GF3 in the fructo-oligosaccharide preparations were small, and the concentration in the sample was low. The PLS-1 model was calibrated using the sugar concentration as the response variable, which was measured by HPLC, as in the case of enzyme-treated orange juice. The wavenumber selection was verified only by absorbance, and the calibrated model was evaluated by full cross-validation due to the small number of samples. The wavenumbers extracted from the absorbance spectra of GF4 were 9 points.

**Figure 7:**
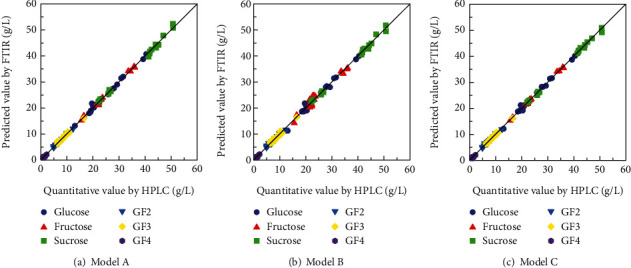
Relationship between predictive values by FTIR and quantitative values by HPLC of sugars in citrus fruit juice containing fructo-oligosaccharides. Model A: *R*^2^ of glucose, fructose, sucrose, GF2, GF3, and GF4 were 0.992, 0.996, 0.995, 0.990, 0.983, and 0.743, respectively, and the number of factors was 5, 5, 5, 6, 6, and 6, respectively. Model B: *R*^2^ of glucose, fructose, sucrose, GF2, GF3, and GF4 were 0.986, 0.968, 0.996, 0.963, 0.971, and 0.724, respectively, and the number of factors was 5, 3, 5, 5, 7, and 7, respectively. Model C: *R*^2^ of glucose, fructose, sucrose, GF2, GF3, and GF4 were 0.994, 0.998, 0.998, 0.979, 0.987, and 0.685, respectively, and the number of factors was 5, 5, 5, 5, 7, and 6, respectively.

**Figure 8:**
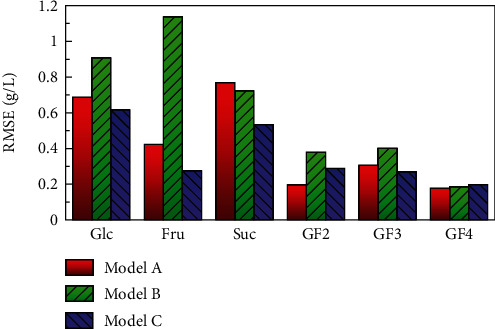
Effect of wavenumber selection step on RMSE of citrus fruit juice containing fructo-oligosaccharides.

Figures 7(a)–7(c) show the comparisons between the predicted sugar concentration values obtained by cross-validation after selecting for wavenumbers in the patterns from A to C and the quantitative values measured by HPLC. Predictions for glucose, fructose, sucrose, GF2, and GF3 could be made with high accuracy with a coefficient of determination of 0.95 or higher as well as GF4 with a coefficient of determination of 0.68. Since the concentration of GF4 was about 1 g/L and the measurement accuracy was inferior under the HPLC conditions, it was assumed that the quantification accuracy by PLS was also inferior to that of other components.  Figure 8 shows the RMSE of predicted values A to C. The absorption tendencies of fructo-oligosaccharides added fruit juice and enzymatically treated orange juice were similar, and the accuracy of concentration prediction was low in model B, which uses only a single component information; however, the error was reduced by using the information from multicomponents. Model C produced equivalent or better results to model A using the full spectrum from 1250 to 900 cm^−1^, and it was possible to predict the concentration with results comparable to that of HPLC. Even in various fruit juices containing GF2, GF3, and GF4, it was possible to reduce the wavenumbers by focusing on the fundamental vibrations of the contained components.

## 4. Conclusions

Two separate methods, i.e., the SE and PLS methods, were compared to predict the sugar components in enzyme-treated orange juice. The PLS method predicted the sugar components with the same accuracy as HPLC; however, overfitting could not be avoided since too much wavenumber information was used. The SE method uses only the wavenumber information for the number of components it contains, but it is unsuitable within the low concentration range. In addition, a simple information is insufficient in predicting sugar components in samples where multiple components are mixed in a complex manner.

The wavenumbers used for model calibration could be significantly reduced by selecting those from the wavenumber and assignment information of the component. By selecting the wavenumber based on the information from the structures of multiple components, it was evident that, even with limited wavenumbers, it was possible to perform model calibrations capable of predicting concentrations with an accuracy equal to or higher than that obtained using the full spectrum. The developed method could be applicable to various biological reaction systems involving complicated mixtures of organic matters with similar structures, for example, for in-line monitoring of sugar concentrations during enzymatic reactions and fermentation processes.

## Figures and Tables

**Table 1:** Sugar concentrations of citrus fruit juices with added fructo-oligosaccharides as determined by HPLC.

		Concentrations (g/L)					
		Glc	Fru	Suc	GF2	GF3	GF4
Valencia orange	A	18.5	21.6	41.3	5.0	6.4	1.0
	B	19.0	21.8	41.7	6.6	8.5	0.7
	C	19.2	22.0	41.8	8.2	10.9	1.5
	D	19.6	22.3	42.5	12.4	16.5	2.2
	E	25.8	22.7	44.2	8.1	8.0	1.0
	F	27.7	23.1	45.2	10.0	10.2	1.4
	G	30.8	33.6	22.0	4.9	6.4	1.0
	H	31.6	34.4	22.6	6.6	8.7	0.9
	I	39.3	36.0	25.9	8.2	8.2	1.1
	J	40.4	35.8	26.2	10.0	10.3	1.4
Mandarin orange	K	20.4	22.3	50.8	5.8	7.6	1.0
	L	20.2	22.2	50.6	7.5	10.0	1.4
Citrus unshiu	M	13.1	15.6	44.0	6.8	8.9	1.2
	N	20.1	16.5	47.0	8.3	8.4	1.1
White grapefruit	O	20.8	21.6	22.2	5.6	7.5	1.0
	P	28.1	23.6	26.0	7.0	7.1	0.9
Ruby grapefruit	Q	20.8	21.5	22.9	7.3	9.8	1.3
	R	29.3	23.2	26.8	9.1	9.2	1.3

## Data Availability

The data used to support the findings of this study are available from the corresponding author upon request.

## Supplementary Materials

Supplementary Materials Table S1 (a, b) shows the wavenumbers used as explanatory variables in the PLS model calibration for models A to C for enzymatically treated orange juice. Table S2 shows the wavenumbers used as explanatory variables in the PLS model calibration for models A to C for fruit juice with added fructo-oligosaccharides. Table S3 shows sugar concentrations of citrus fruit juices with added fructo-oligosaccharides as determined by PLS.
