# Supplementary material for: Simultaneous Content Determination of Mono-, Di-, and Fructo-oligosaccharides in Citrus Fruit Juices Using an FTIR-PLS Method Based on Selected Absorption Bands
Source: Int J Food Sci. 2024 Jan 10;2024:9265590. doi: 10.1155/2024/9265590 (PMC10794075; doi:10.1155/2024/9265590)
Supplement: Supplementary Materials — Table S1 (a, b) shows the wavenumbers used as explanatory variables in the PLS model calibration for models A to C for enzymatically treated orange juice. Table S2 shows the wavenumbers used as explanatory variables in the PLS model calibration for models A to C for fruit juice with added fructo-oligosaccharides. Table S3 shows sugar concentrations of citrus fruit juices with added fructo-oligosaccharides as determined by PLS. [file 9265590.f1.pdf]

Supplementary Materials

Table S1. Wavenumbers used in the model calibration of enzymatically treated orange juice

(a) Absorbance

	Wavenumber (cm^{-1})				
	**Glc**	**Fru**	**Suc**	**GF2**	**GF3**
**Model A**	900, 902, 904, 906, 908, 910, 912, 914, 916, 918, 920, 922, 924, 926, 928, 930, 932, 934, 936, 938, 940, 942, 944, 946, 948, 950, 952, 954, 956, 958, 960, 962, 964, 966, 968, 970, 972, 974, 976, 978, 980, 982, 984, 986, 988, 990, 992, 994, 996, 998, 1000, 1002, 1004, 1006, 1008, 1010, 1012, 1014,1016, 1018, 1020,1022, 1024, 1026, 1028, 1030, 1032, 1034, 1036, 1038, 1040, 1042, 1044, 1046, 1048, 1050, 1052, 1054, 1056, 1058, 1060, 1062, 1064, 1066, 1068, 1070, 1072, 1074, 1076, 1078, 1080, 1082, 1084,1086, 1088, 1090, 1092, 1094, 1096, 1098, 1100, 1102, 1104, 1106, 1108, 1110, 1112, 1114, 1116, 1118,1120, 1122, 1124, 1126, 1128, 1130, 1132, 1134, 1136, 1138, 1140, 1142, 1144, 1146, 1148, 1150, 1152, 1154, 1156, 1158, 1160, 1162, 1164, 1166, 1168, 1170, 1172, 1174, 1176, 1178,1180, 1182, 1184, 1186, 1188, 1190, 1192, 1194, 1196, 1198, 1200, 1202, 1204,1206, 1208, 1210, 1212, 1214, 1216, 1218, 1220, 1222, 1224, 1226, 1228, 1230, 1232, 1234, 1236, 1238, 1240, 1242, 1244, 1246, 1248, 1250				
**Model B**	918, 994, 1018, 1034, 1056, 1080, 1106, 1152	920, 968, 980, 1018, 1034, 1064, 1084, 1102, 1156, 1182	928, 1000, 1018, 1032, 1056, 1082, 1114, 1138, 1162, 1212	930, 1002, 1020, 1034, 1056, 1078, 1116, 1136, 1162, 1216	930, 998, 1018, 1034, 1054, 1080, 1116, 1136, 1162, 1220
**Model C**	918, 920, 928, 930, 968, 980, 994, 998, 1000, 1002, 1018, 1020, 1032, 1034, 1054, 1056, 1064, 1078, 1080, 1082, 1084, 1102, 1106, 1114, 1116, 1136, 1138, 1152, 1156, 1162, 1182, 1212, 1216, 1220				

(b) 2nd derivative

	Wavenumber (cm^{-1})				
	**Glc**	**Fru**	**Suc**	**GF2**	**GF3**
**Model A**	900, 902, 904, 906, 908, 910, 912, 914, 916, 918, 920, 922, 924, 926, 928, 930, 932, 934, 936, 938, 940, 942, 944, 946, 948, 950, 952, 954, 956, 958, 960, 962, 964, 966, 968, 970, 972, 974, 976, 978, 980, 982, 984, 986, 988, 990, 992, 994, 996, 998, 1000, 1002, 1004, 1006, 1008, 1010, 1012, 1014,1016, 1018, 1020,1022, 1024, 1026, 1028, 1030, 1032, 1034, 1036, 1038, 1040, 1042, 1044, 1046, 1048, 1050, 1052, 1054, 1056, 1058, 1060, 1062, 1064, 1066, 1068, 1070, 1072, 1074, 1076, 1078, 1080, 1082, 1084,1086, 1088, 1090, 1092, 1094, 1096, 1098, 1100, 1102, 1104, 1106, 1108, 1110, 1112, 1114, 1116, 1118,1120, 1122, 1124, 1126, 1128, 1130, 1132, 1134, 1136, 1138, 1140, 1142, 1144, 1146, 1148, 1150, 1152, 1154, 1156, 1158, 1160, 1162, 1164, 1166, 1168, 1170, 1172, 1174, 1176, 1178,1180, 1182, 1184, 1186, 1188, 1190, 1192, 1194, 1196, 1198, 1200, 1202, 1204,1206, 1208, 1210, 1212, 1214, 1216, 1218, 1220, 1222, 1224, 1226, 1228, 1230, 1232, 1234, 1236, 1238, 1240, 1242, 1244, 1246, 1248, 1250				
**Model B**	920, 990, 1034, 1080, 1110, 1156	922, 974, 1062, 1088, 1108, 1156, 1188	928, 996, 1056, 1110, 1140, 1212	928, 994, 1028, 1060, 1074, 1114, 1136, 1168, 1216	930, 994, 1030, 1060, 1072, 1114, 1138, 1168, 1218
**Model C**	920, 922, 928, 930, 974, 990, 994, 996, 1028, 1030, 1034, 1056, 1060, 1062, 1072, 1074, 1080, 1088, 1108, 1110, 1114, 1136, 1138, 1140, 1156, 1168, 1188, 1212, 1216, 1218				

Table S2. Wavenumbers used in the model calibration of fruit juice with added fructo-oligosaccharides.

	Wavenumber (cm^{-1})					
	**Glc**	**Fru**	**Suc**	**GF2**	**GF3**	**GF4**
**Model A**	900, 902, 904, 906, 908, 910, 912, 914, 916, 918, 920, 922, 924, 926, 928, 930, 932, 934, 936, 938, 940, 942, 944, 946, 948, 950, 952, 954, 956, 958, 960, 962, 964, 966, 968, 970, 972, 974, 976, 978, 980, 982, 984, 986, 988, 990, 992, 994, 996, 998, 1000, 1002, 1004, 1006, 1008, 1010, 1012, 1014,1016, 1018, 1020, 1022, 1024, 1026, 1028, 1030, 1032, 1034, 1036, 1038, 1040, 1042, 1044, 1046, 1048, 1050, 1052, 1054, 1056, 1058, 1060, 1062, 1064, 1066, 1068, 1070, 1072, 1074, 1076, 1078, 1080, 1082, 1084, 1086, 1088, 1090, 1092, 1094, 1096, 1098, 1100, 1102, 1104, 1106, 1108, 1110, 1112, 1114, 1116, 1118,1120, 1122, 1124, 1126, 1128, 1130, 1132, 1134, 1136, 1138, 1140, 1142, 1144, 1146, 1148, 1150, 1152, 1154, 1156, 1158, 1160, 1162, 1164, 1166, 1168, 1170, 1172, 1174, 1176, 1178,1180, 1182, 1184, 1186, 1188, 1190, 1192, 1194, 1196, 1198, 1200, 1202, 1204, 1206, 1208, 1210, 1212, 1214, 1216, 1218, 1220, 1222, 1224, 1226, 1228, 1230, 1232, 1234, 1236, 1238, 1240, 1242, 1244, 1246, 1248, 1250					
**Model B**	918, 994, 1018, 1034, 1056, 1080, 1106, 1152	920, 968, 980, 1018, 1034, 1064, 1084, 1102, 1156, 1182	928, 1000, 1018, 1032, 1056, 1082, 1114, 1138, 1162, 1212	930, 1002, 1020, 1034, 1056, 1078, 1116, 1136, 1162, 1216	930, 998, 1018, 1034, 1054, 1080, 1116, 1136, 1162, 1220	932, 996, 1018, 1036, 1054, 1118, 1134, 1162, 1218
**Model C**	918, 920, 928, 930, 932, 968, 980, 994, 996, 998, 1000, 1002, 1018, 1020, 1032, 1034, 1036, 1054, 1056, 1064, 1078, 1080, 1082, 1084, 1102, 1106, 1114, 1116, 1118, 1134, 1136, 1138, 1152, 1156, 1162, 1182, 1212, 1216, 1218, 1220					

Table S3. Sugar concentrations of citrus fruit juices with added fructo-oligosaccharides as determined by PLS

		Concentrations (g/L)					
		Glc	Fru	Suc	GF2	GF3	GF4
Valencia Orange	A	18.8	21.6	41.1	5.1	6.6	0.6
	B	19.0	21.6	41.8	6.3	8.5	1.2
	C	19.2	21.9	42.3	8.0	10.7	1.4
	D	21.2	21.9	43.3	12.0	16.2	2.1
	E	26.1	22.9	44.4	8.1	8.4	1.0
	F	28.3	23.5	45.5	9.9	9.8	1.3
	G	31.4	34.2	22.2	4.8	6.3	0.7
	H	31.6	34.3	22.8	6.4	8.4	1.2
	I	38.7	35.5	25.1	8.2	8.1	1.1
	J	40.2	35.8	26.4	10.4	10.8	1.4
Mandarin Orange	K	21.2	21.8	22.5	5.7	7.7	1.0
	L	28.2	23.5	25.9	6.7	6.8	0.9
Citrus Unshiu	M	20.7	21.5	23.1	7.4	9.8	1.3
	N	28.7	23.2	26.3	9.2	9.4	1.4
White Grapefruit	O	12.2	15.3	43.3	7.1	9.2	1.2
	P	20.2	16.6	46.9	8.3	8.1	1.1
Ruby Grapefruit	Q	19.2	21.8	49.2	6.6	7.9	1.1
	R	20.1	22.1	50.9	7.7	9.8	1.2
